# *In situ* transformation of CuBi_2_O_4_ to heterogeneous Bi_2_O_3_/Cu_2_O mixed phase using thermal method for exceptional H_2_ production

**DOI:** 10.1039/d6ra07504e

**Published:** 2026-10-23

**Authors:** Indu Kumari, Hazina Charles, Plassidius J. Chengula, Vijay Kumar, Siddhant N. Deshpande, Caroline S. Lee, Rajendra C. Pawar

**Affiliations:** a Department of Physics, Central University of Rajasthan Ajmer India rajendra.pawar@curaj.ac.in; b Department of Materials and Chemical Engineering, Hanyang University ERICA Campus Ansan South Korea sunyonglee@hanyang.ac.kr

## Abstract

Photoelectrochemical water splitting using semiconductor materials is a viable approach to produce green hydrogen (H_2_). CuBi_2_O_4_ is a promising option because of its high redox potential and advantageous band gap; however, its use is hindered by significant charge carrier recombination and inadequate interfacial contact. We synthesized CuBi_2_O_4_ using a simple thermal method and examined the impact of polyvinyl alcohol on its structural and morphological characteristics. We observed a phase transformation from a mixed-phase CuBi_2_O_4_ to a highly efficient α-Bi_2_O_3_/β-Bi_2_O_3_/Cu_2_O heterostructure. The optimized heterostructure (α-Bi_2_O_3_/β-Bi_2_O_3_/Cu_2_O) showed a remarkable photocatalytic H_2_ evolution rate of 776 µmol g^−1^ compared with the pristine (CuBi_2_O_4_) structure, which achieved a rate of 166 µmol g^−1^. The exceptional photocatalytic H_2_ production of the heterogeneous sample arises from the efficient charge transfer mechanism and separation of photogenerated electron–hole pairs facilitated by the interfaces produced between the distinct phases. The enhanced transient photoresponse of α-Bi_2_O_3_/β-Bi_2_O_3_/Cu_2_O (9.76 µA) compared with that of the pure sample (5.13 µA) validates the effective generation and separation of photogenerated charge carriers resulting from the appropriate band alignment and *in situ* p–n junctions formed during the synthesis process. Furthermore, the electrochemical impedance spectra showed a reduced charge transfer resistance for the optimized heterostructure compared with that of the pure structure, thereby validating the superior photocatalytic H_2_ evolution of the heterostructure. Consequently, using the thermal approach establishes a simple synthesis route for heterogeneous catalysts with exceptional H_2_ evolution performance. Moreover, this study advances the development of H_2_ technology by providing a cost-effective strategy for designing efficient catalysts.

## Abstract 2

CuBi_2_O_4_ synthesis *via* a thermal method and its H_2_ evolution performance with the proposed energy band diagram.

## Graphical abstract

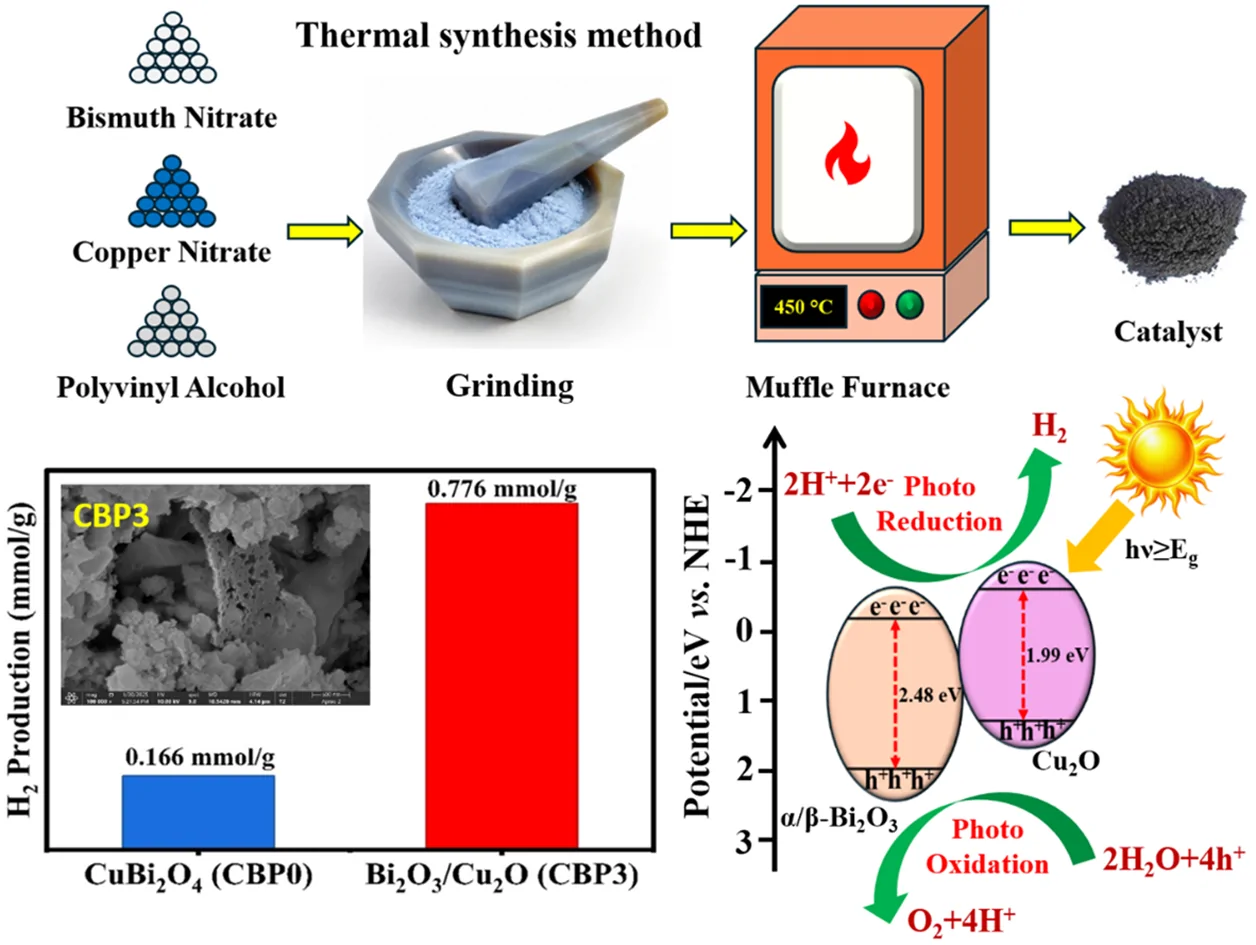

## Introduction

The application of semiconducting materials for photoelectrochemical (PEC) water splitting into hydrogen (H_2_) and oxygen (O_2_) gases is a viable approach to meet increasing energy demands.^1^ It is one of the most effective approaches for mitigating the adverse impacts of climate change and meeting the rising energy demand. Its efficiency depends on the catalyst used to facilitate the reaction under solar light. Several factors such as light absorption capacity, crystalline structure, redox potential, charge carrier dynamics, thermal and chemical stability, and type of heterostructures play crucial roles in achieving the desirable performance.^2–5^ Various nanostructures such as binary oxides (TiO_2_, ZnO, WO_3_, and SnO_2_), ternary oxides (BiVO_4_, Bi_2_WO_6_ and Bi_2_MoO_6_), chalcogenides (MoS_2_, CdS, and ZnS), and perovskites (SrTiO_3_, BaTiO_3_, and LaFeO_3_) have been explored as photocatalysts.^6–13^ The incorporation of these nanostructures into a singular multifunctional nanocomposite continues to be a primary objective for achieving holistic and energy-efficient PEC water splitting and degradation. CuBi_2_O_4_ (CBO) is an interesting candidate owing its narrow band gap (1.5–1.8 eV) ensuring visible light absorption and more negative conduction band edge than the reduction potential of H^+^/H_2_, which meets the criteria for water splitting reaction.^14,15^ CBO, which is a p-type semiconductor, crystallizes in a tetragonal kusachiite structure.^16^ It is nontoxic and earth-abundant, thereby making it desirable for green PEC H_2_ generation. However, CBO suffers from several drawbacks including high charge carrier recombination rate, high charge transfer resistance, and poor photostability.^17,18^ Several strategies such as doping, co-catalyst loading, heterojunctions, and surfactant addition have been employed to increase the efficiency of CBO.^19–21^ In particular, the use of surfactants during the growth process is an effective strategy for inducing morphological and chemical modifications. Various surfactants such as sodium dodecyl sulfate, pluronic 123, cetyltrimethylammonium bromide (CTAB), polyvinyl alcohol (PVA), polyvinyl pyrrolidone, trisodium citrate and polyethylene glycol (PEG-400) have been employed, which form different morphologies such as nanoparticles, hedgehog like microspheres, and hierarchical nanoflowers.^22–26^ According to the literature, desirable morphologies increase the surface area, which leads to more active sites for catalytic reactions.^27^ Specifically, PVA is recognized for its relative thermostability, non-toxicity, and its role as a polymer matrix during the growth of nanostructures.^28^ The plentiful hydroxyl (–OH) functional groups along the PVA backbone exhibit selective coordination with particular crystal planes of developing structures. Therefore, it can be effectively employed to regulate morphology, regulate particle size, prevent agglomeration resulting in crystalline phase formation, and facilitate surface chemical modifications.^29–31^ Another strategy to improve the catalytic performance is the formation of heterojunctions, such as type-II and S schemes, with other suitable semiconductors. These heterojunctions have improved charge carrier separation and higher redox potential, thereby leading to improved performance compared with only CBO.^32–36^ However, PEC H_2_ production of current CBO-based heterojunctions is inadequate for their use in commercial applications. Accordingly, additional measures are essential to further enhance the performance.

In this study, we synthesized mixed-phase CBO using a single-step thermal method and subsequently analyzed the effect of polyvinyl alcohol (PVA) incorporation during the synthesis on the structural and morphological development. The ideal introduction of PVA resulted in a phase transition from the mixed-phase CBO to an *in situ* α-Bi_2_O_3_/β-Bi_2_O_3_/Cu_2_O heterostructure, which can be attributed to the effect of PVA on the diffusion of Cu^2+^ and Bi^3+^ ions during thermal processing and structural development. The heterojunction exhibited a considerable enhancement in water splitting efficiency relative to the pristine structure, which can be attributed to the efficient charge separation and superior interfacial contact resulting from the *in situ* synthesis of the heterojunction. The transient photoresponse validated the substantial reduction in the charge transfer resistance of the heterojunction. Consequently, employing a simple thermal synthesis method with the incorporation of PVA is a proficient technique for producing highly effective *in situ* heterostructures.

## Experimental details

### Materials

Bi (NO_3_)_3_·5H_2_O (98%), Cu (NO_3_)_2_·3H_2_O (95–103%), and PVA were purchased from Qualigens.

### Thermal CBO synthesis

The synthesis methodology is shown in Fig. 1. Bi(NO_3_)_3_·5H_2_O and Cu(NO_3_)_2_·3H_2_O are used as the initial precursors for the synthesis of the CBO nanostructures. In the first step, 0.605 g of Bi(NO_3_)_3_·5H_2_O and 0.150 g of Cu(NO_3_)_2_·3H_2_O are ground using a mortar pestle for 1 h. Subsequently, the mixture is transferred into a ceramic crucible and sintered in a muffle furnace at 450 °C for 1 h at a ramping rate of 2 °C min^−1^. After the sintering, the powder is ground for 2 min and collected. To enable structural modification and formation of different phases, PVA is introduced as a surfactant during the initial grinding step.

**Fig. 1:**
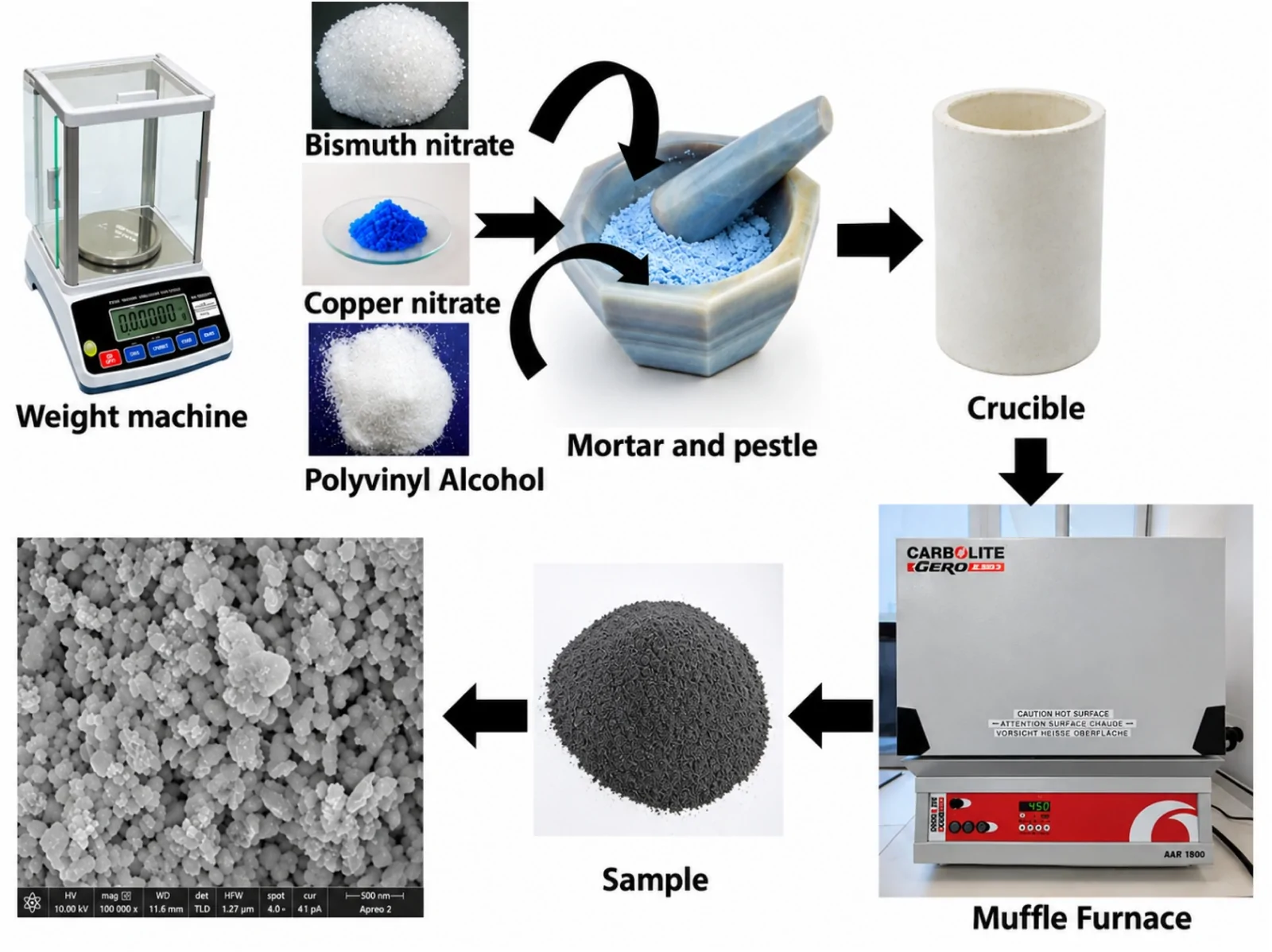
Synthesis methodology using a thermal method.

Varying amounts of PVA, *i.e.*, 0.218 g, 0.436 g, 0.80 g, 1.30 g, 1.70 g, and 2.10 g, yield different samples, which are labelled as CBP0, CBP1, CBP2, CBP3, CBP4, CBP5, and CBP6, respectively.

### Measurements

The morphologies, sizes, and microstructures of the structures were examined using field emission scanning electron microscopy (FE-SEM; Apreo 2S; Thermo Fisher Scientific, UK & Hitachi s4800 SEM) and transmission electron microscopy (TEM; 200 kV; JEM2100F; JEOL, Japan). Crystallinity and orientation were examined using X-ray diffraction (XRD; D/Max-2500/PC; Cu Kα source; *λ* = 1.5418 Å; Rigaku, Japan). Using a dry-press disk and BaSO_4_ as the background, changes in the optical properties were measured using ultraviolet-visible spectrophotometry (V-650; Jasco, Japan) within a wavelength range of 200–900 nm. The chemical composition, valence states, and band configurations of the materials were characterized using X-ray photoelectron spectroscopy (XPS; K-Alpha+; Al Kα source; Thermo Fisher Scientific, UK). The TR-PL study was carried out using a confocal microscope (MicroTime-200, Picoquant, Germany). A single-mode pulsed diode laser (375 nm) was used as an excitation source. Exponential function fitting for the obtained emission decays was accomplished using the Symphotime-64 software (Ver. 2.2) by the exponential decay model. Steady-state PL spectra were recorded by a fluorescence spectrophotometer (F-7000, Hitachi).

### PEC measurements

Transient photocurrent response (PC) spectra and electrochemical impedance spectroscopy (EIS) data were captured using an electrochemical workstation (VersaSTAT 4, Princeton Applied Research). Visible light illumination was used, and a standard three-electrode setup was employed. The working electrodes were prepared using each sample (CBP0–CBP6), with a Pt wire as the counter electrode, Ag/AgCl as the reference electrode, and 0.5 M aqueous Na_2_SO_4_ solution as the electrolyte.

### Photocatalytic water splitting measurements

A specially designed quartz reactor was used to assess the photocatalytic water splitting (H_2_) performances of CBP0 and CBP3 structures. In a typical photocatalytic reactor, 100 mL of an aqueous solution of 10 vol% triethanolamine (TEOA) was incorporated with 20 mg of the catalyst while being continuously stirred. The surface of the suspended photocatalyst was then sensitized to a Pt (1 weight percent) cocatalyst using a UV reduction technique. To get rid of the dissolved oxygen (O), nitrogen (N) was added to the suspension. After that, a photocatalytic reactor was used to irradiate the purged solution. To fully remove the air, the reactor was then vacuum-purged with argon gas for 30 minutes. A 300 W Xe lamp equipped with a 400 nm cut-off filter was used to irradiate the vacuumed suspension for six hours. A gas chromatograph (model 6500 GC; YL instruments) with a thermal conductivity detector (TCD) was used to measure the evolved H_2_ gas (150 µl), which was collected using a syringe at intervals of one hour.

### Reaction mechanism

The synthesis of CBO includes multiple chemical and thermal transformations, which affect the phase formation and morphology. In the first step, grinding ensures homogeneous mixing and may lead to minor interactions such as H_2_ bonding and partial decomposition. The main phase formation occurs during the sintering step, where the nitrates decompose thermally and copper and bismuth ions react and form the CBO phase. The introduction of PVA before grinding changes how the reaction proceeds. In the grinding step, PVA acts as a surfactant and adsorbs onto the precursor particles, thereby affecting the contact and diffusion between Cu^2+^ and Bi^3+^ ions and achieving stearic stabilization.^31,37^ The presence of PVA reduces the interaction between these ions, thereby lessening the agglomeration and adherence of ions with each other, which leads to the improved morphology and impacts phase formation.^38,39^ According to the literature,^40–42^ decomposition of PVA begins at 200 °C with approximately 3% loss, and the majority, around 83%, is decomposed by 400 °C. Below 400 °C, the removal of water and acetic acid from PVA, along with the oxidation of vinyl chains, has led to the majority of weight loss. The continued rise in sintering temperature leads to a reduction in the weight loss rate while concurrently initiating the formation of carbonaceous residue within the sample. In this study, the sintering process at 450 °C for 1 hour likely resulted in the decomposition of the majority of the PVA content, potentially leaving behind a small percentage of carbonaceous residue. Further, during sintering, PVA decomposes and releases gases, thereby affecting the microstructure and phase evolution of CBO.^43^

Moreover, optimal use of PVA resulted in enhanced morphology due to a uniform distribution of particles and regulated nucleation. Under these conditions, the final sample comprises both α and β phases of Bi_2_O_3_ in addition to Cu_2_O. Excessive PVA can hinder ion diffusion and lead to the development of secondary impurity phases like CuO and Bi, or contribute to an increased presence of the less efficient α-Bi_2_O_3_ phase. Consequently, the reaction mechanism is significantly influenced by the presence of PVA, which governs the structure, morphology, and phase of the synthesized photocatalysts.

## Results and discussion

### XRD analysis

The XRD analysis patterns shown in Fig. 2A reveal that as the PVA content increases during synthesis, the crystalline phase undergoes significant change. Without PVA addition (CBP0), CBO is the primary phase, with peaks corresponding to the kusachiite structure. Major diffraction peaks are observed at 2θ values of 27.91°, 30.60°, 34.22°, 46.72°, and 55.64°, which correspond to the (211), (002), (112), (411), and (332) planes of CBO, respectively. Additionally, few minor peaks corresponding to Bi_2_O_3_ and CuO phases are present, which confirm the presence of these secondary phases.

**Fig. 2:**
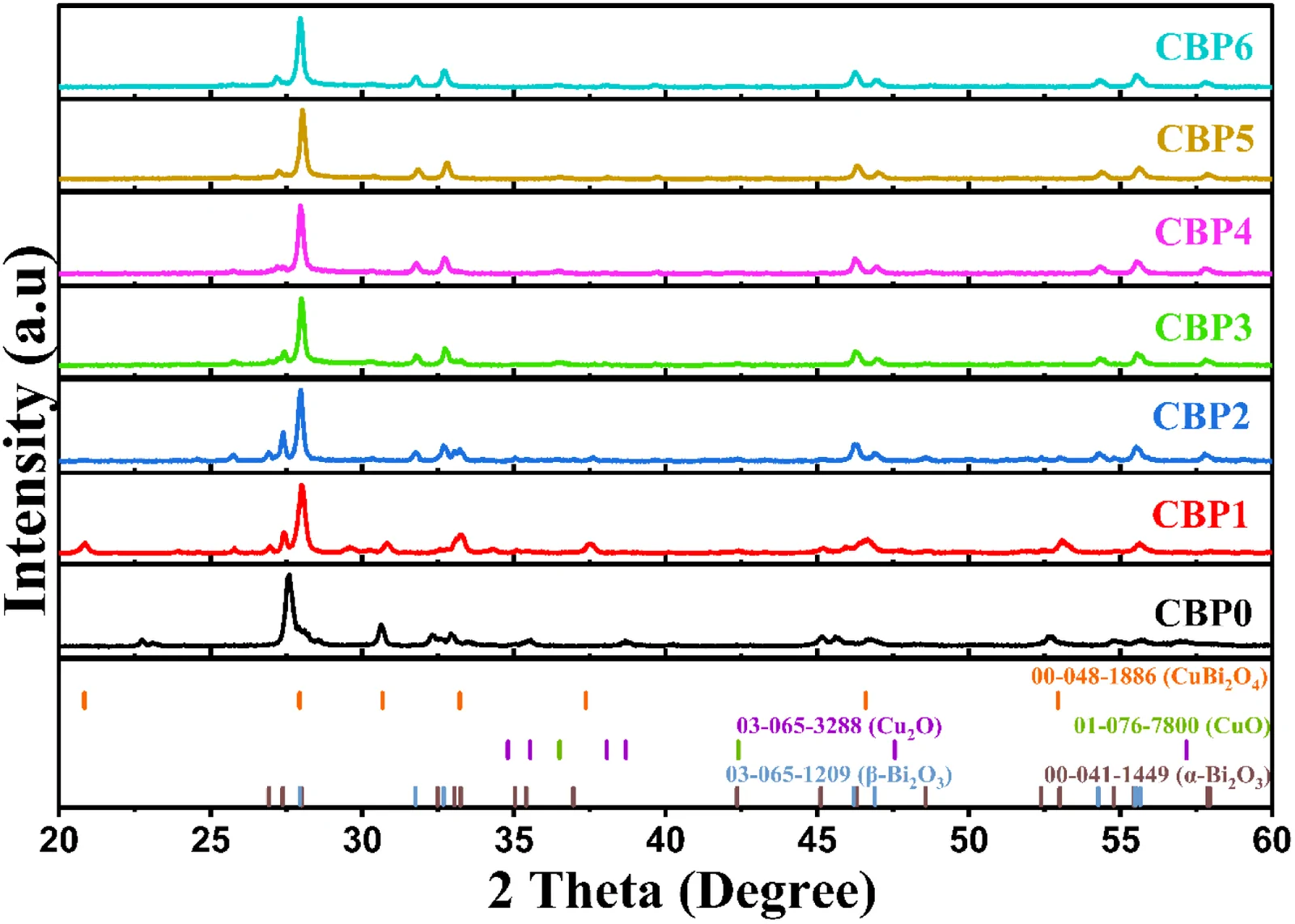
XRD patterns of the as-prepared samples CBP0–CBP6.

With the addition of PVA, the CBO peaks gradually decrease. Simultaneously, the intensity of the α-Bi_2_O_3_ and β-Bi_2_O_3_ peaks increases. In CBP1, the CBO phase is still dominant but shows a reduced intensity. On further increasing the PVA content (CBP2 onwards), the XRD patterns largely show α-Bi_2_O_3_ and β-Bi_2_O_3_ reflections. In CBP3, peaks of Cu_2_O appear at 2*θ* = 36.39° and 61.48°, corresponding to the (111) and (220) planes, respectively. This confirms that an α-Bi_2_O_3_/β-Bi_2_O_3_/Cu_2_O heterostructure is formed *via in situ* phase transformation. In Fig. 2A, CBP4–CBP6 show mostly Bi_2_O_3_ peaks. These observations suggest that the use of PVA as an additive induces a phase transformation from the mixed-phase CBO to an α-Bi_2_O_3_/β-Bi_2_O_3_/Cu_2_O heterostructures.

The crystallite sizes were calculated using the Debye–Scherrer formula and are provided in Table 1. The crystal size is found to be increased for in presence of PVA as compared to pristine sample. The increased crystal size could be aroused from the presence of different growth mechanism of nanostructures. As per the literature, there are fundamentally two types of competing growth processes occurring in the presence of PVA: the first is agglomeration growth, which occurs atom-by-atom, and the second is surface integration growth, which takes place particle-by-particle.^44,45^ In the present work, the results suggest that surface integration growth occurs through the collision and fusion of primary particles, leading to the formation of polycrystalline and multi-phase structures. Consequently, the crystallite size is observed to increase progressively with the amount of PVA. Interestingly, the crystallite size of CBP3 exceeds that observed with a higher introduction of PVA. This could originate from the existence of multiple phases within a single cluster throughout the growth process. For the CBP3 sample, we are determining the crystallite size for the Bi_2_O_3_ and Cu_2_O phases in comparison to the pristine sample and other heterostructures. There is also the possibility to affect the crystallite size as a consequence of the interaction between the developing crystallite and PVA molecules throughout the growth process. Therefore, the crystallite size observed in this work appears to be unusual.

**Table 1:** Crystallite sizes of the samples

Sample	CBP0	CBP1	CBP2	CBP3	CBP4	CBP5	CBP6
Crystallite size (Å)	249.60	280.79	374.13	364.61	352.81	353.73	351.93

Furthermore, the XRD patterns of the different acquired samples, including the optimized sample post-stability cycles, were analyzed by Rietveld refinement (Fig. S1), and the phase composition of the samples together with the *χ*^2^ values is included in Table TS1. The *χ*^2^ < 5 indicates a strong correlation between the computed data and the empirical data. Evidently, the phases have been transformed to β-Bi_2_O_3_ (63.8%), α-Bi_2_O_3_(21.2%), and Cu_2_O (14.9%) for CBP_3_ in contrast to the unmodified CBP0 sample. The phase transition verifies that the CBO phase has been converted into heterogeneous Bi_2_O_3_ and Cu_2_O phases.

### FE-SEM analysis

We studied the morphologies of the CBO particles and transformed Bi_2_O_3_/Cu_2_O heterostructure formed by PVA addition using FE-SEM analysis, and the results are shown Fig. 3A–G. As discussed earlier, PVA affects the reaction mechanism during the synthesis, leading to a distinct morphology of the final product. Without PVA, well-defined nanoparticles of CBO which are randomly dispersed are obtained, because the thermal treatment causes crystallization. A few bulky sheets are also observed, leading to irregular morphology overall. The addition of PVA during grinding partially inhibits the interaction of the metal precursors. This modifies the nucleation and growth processes during sintering, thereby leading to changes in the morphology. At the optimal PVA concentration, the material develops unique sheet-like or mesh-like surface structures because PVA decomposes and acts as a temporary template. The modification in the morphology can also be ascribed to PVA-induced phase transformation from CuBi_2_O_4_ to heterogeneous Bi_2_O_3_/Cu_2_O phase. Basically, the precursor residue after mortar pestle exhibits a significant interaction where the large number of hydroxyl (–OH) functional groups of the PVA chains coordinate with the Cu^2+^ and Bi^3+^ cations, thereby ensuring a uniform distribution at the molecular level. Upon subsequent calcination, the PVA forms a rigid, cross-linked polymeric matrix that entraps these metal ions. This network imposes severe spatial constraints, limiting the isotropic diffusion and agglomeration of heterostructures nuclei. As the temperature increases, heterostructure nucleation is forced to occur anisotropically along the continuous channels of the polymer framework. The gradual thermal decomposition of the PVA matrix into gaseous byproducts (CO_2_ and H_2_O) leaves behind a replicated, interconnected sheet-like or mesh-like morphology.^46–48^ Therefore, PVA acts as an active mediator in modifying spatial growth processes, controlling the particle growth and determining the features of the final sheet-like structures.

**Fig. 3:**
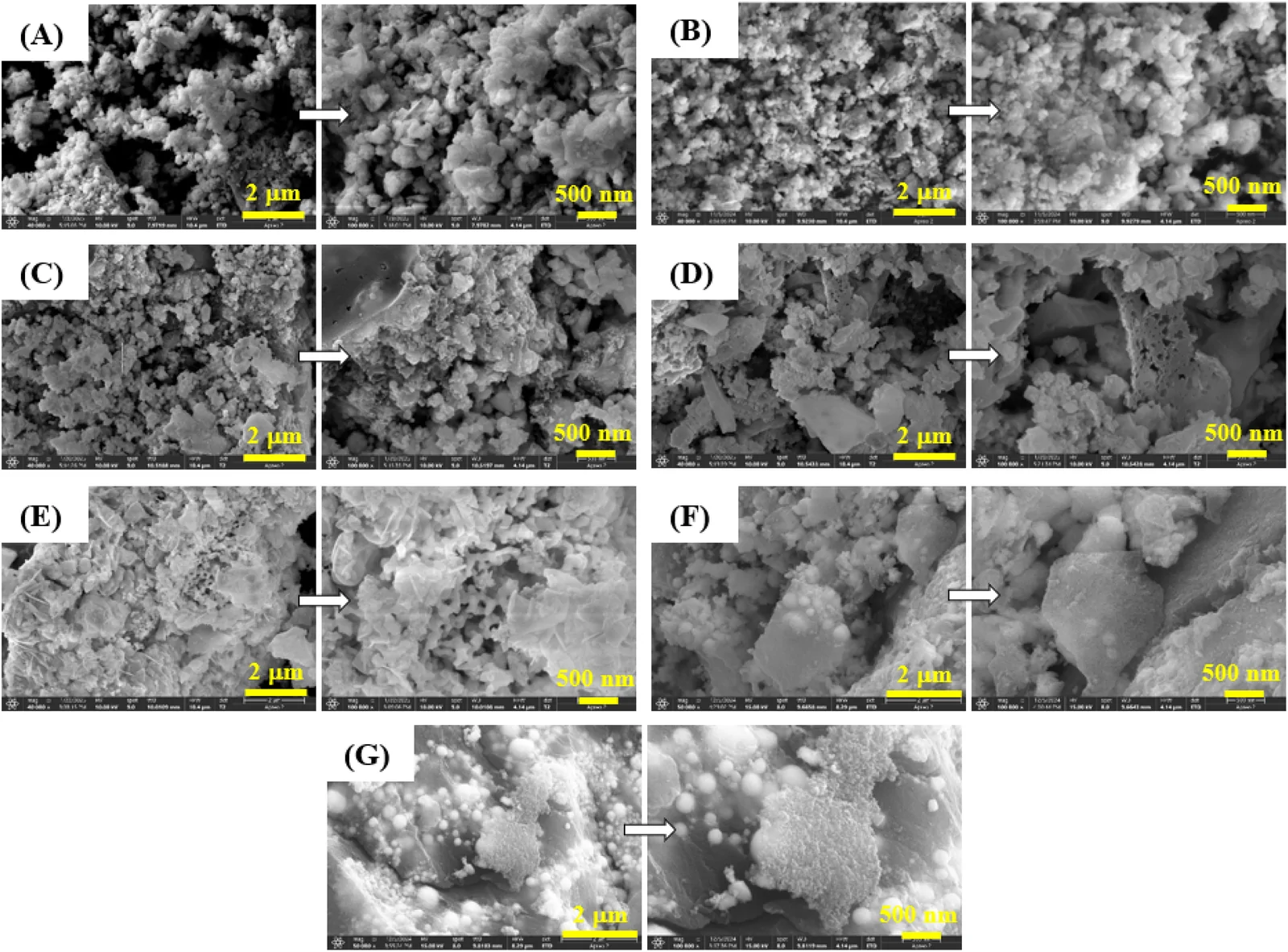
FE-SEM images of (A) CBP0, (B) CBP1, (C) CBP2, (D) CBP3, (E) CBP4, (F) CBP5, and (G) CBP6.

However, excessive PVA disrupts uniform growth and results in irregular structures due to α-Bi_2_O_3_ and Bi phase formation. Thus, by controlling the PVA amount, the surface morphology of CBO can be tailored from agglomerated particles to more porous and structured networks, which are beneficial for enhancing catalytic activity and charge transport in PEC applications.

### TEM analysis

The microstructures of the CBP0 and CBP3 samples were studied using TEM and HRTEM. As shown in Fig. 4A, CBP0 exhibits an irregular morphology consisting of sheets and particles. This could be due to the breakdown of macro-aggregates during sampling, which exposes the underlying nanoscale morphology. In Fig. 4B, multiple orientations of the lattice fringes with interplanar spacings ranging from 0.195 to 0.290 nm are observed. These orientations are caused by the multiphase nature of the sample. Interestingly, the interplanar spacing of 0.290 nm and 0.195 nm is associated with the CBO phase, while 0.270 nm corresponds to the α phase of Bi_2_O_3_, respectively. For CBP3, a distribution of particles of different sizes is observed in Fig. 4C. From Fig. 4D, the interplanar spacing is seen to be 0.271 nm and 0.309 nm, corresponding to α and β phases of Bi_2_O_3_. Fig. 4E and F show the SAED patterns of CBP0 and CBP3, where the diffraction spots are indexed to the lattice planes. In the CBP0 pattern, spots ascribing to the (411) plane of CBO, and (121) plane of α-Bi_2_O_3_ are seen, thereby confirming our observations from the XRD analysis. Similarly, in the CBP3 patterns, spots corresponding to the (121) plane of the α phase and (201) plane of the β phase of Bi_2_O_3_ are seen, along with (111) plane of Cu_2_O, thereby confirming these as the major phases present in the sample.

**Fig. 4:**
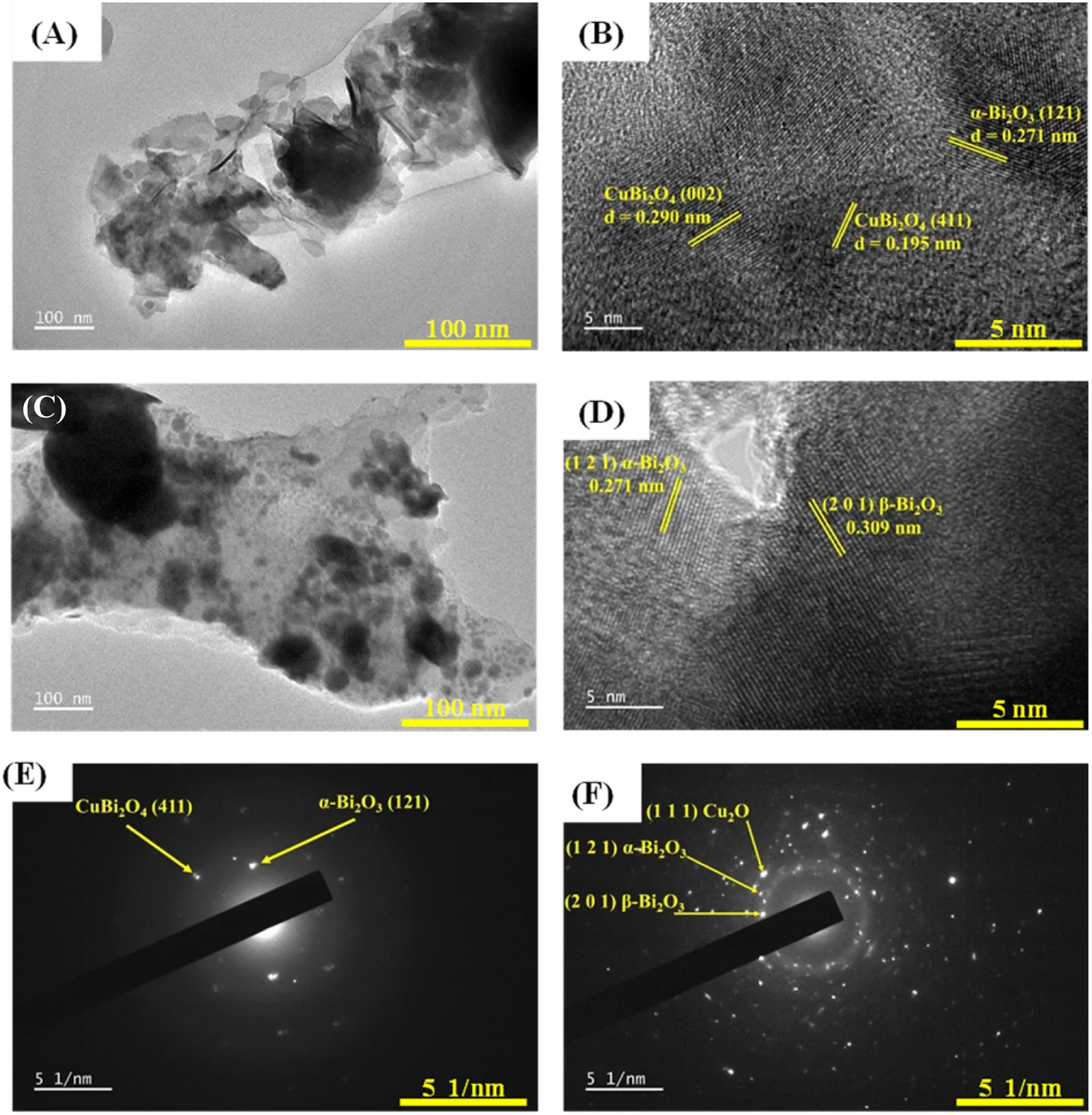
HRTEM images of (A) and (B) CBP0 and (C) and (D) CBP3; SAED patterns of (E) CBP0 and (F) CBP3.

### Band gap estimation

The absorbance spectra of the synthesized samples were obtained, and Tauc plots were used to estimate the band gaps. The Tauc relation correlates absorption coefficient (*α*) and energy of incident photons (*hν*) as,^49^*αhν* = *A*(*hν* − *E*_g_)^*n*^where *A* is a constant, *E*_g_ is the optical bandgap, and *n* decides direct or indirect nature of the optical transition.

Fig. 5A and B show the absorbance spectra and Tauc plots, in which the as-prepared samples exhibit a direct band gap. The band gap values are listed in Table 2. The CBP0 sample exhibits a distinct band gap at 1.62 eV, corresponding to the CBO majority phase, in agreement with the XRD results.^14^ From CBP1 to CBP6, the optical band gaps gradually increase with increasing PVA amount, reaching up to 2.83 eV for CBP6. This widening of the band gap is primarily due to the emergence of the α-Bi_2_O_3_, β-Bi_2_O_3_, and Cu_2_O phases, which have wider individual band gaps than CBO. PVA decomposition possibly leaves behind a carbon residue, which introduces localized states that affect the band structure. These results indicate that the addition of PVA during synthesis has a significant effect on the optical properties of the material.

**Fig. 5:**
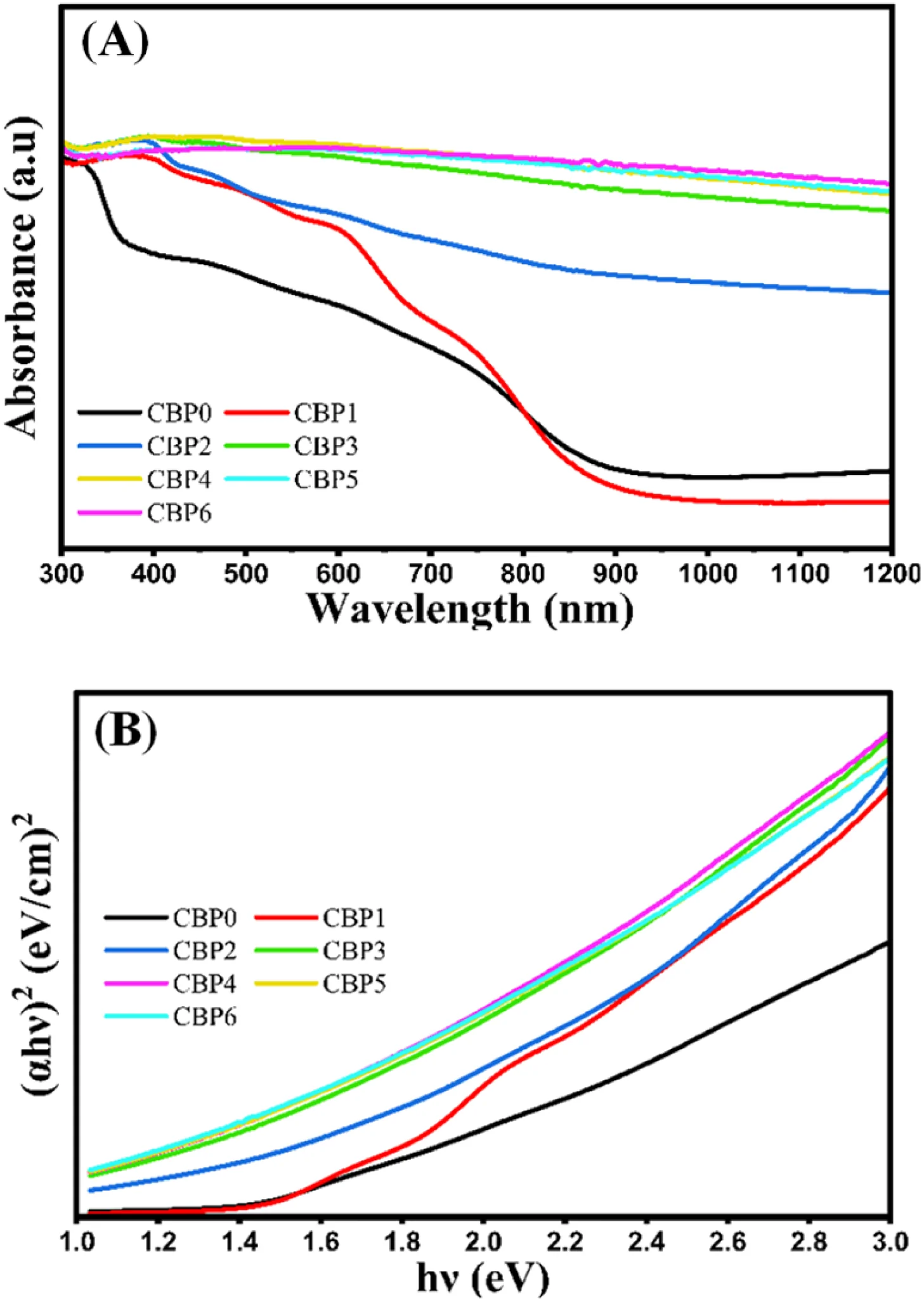
(A) Optical absorbance and (B) Tauc plots of the samples CBP0–CBP6.

**Table 2:** Optical band gap values of the samples

Sample	CBP0	CBP1	CBP2	CBP3	CBP4	CBP5	CBP6
Band gap (eV)	1.62	1.71	2.31	2.38	2.62	2.81	2.83

### XPS analysis

To determine the surface states and elemental composition of the CBP0 and CBP1 samples, XPS spectra were analyzed, and fitting was performed using Kherve Fitting software.^50^ The binding energy was calibrated based on the adventitious carbon peak at 284.8 eV. All the peak positions are listed in Table 3. Fig. 6A and B show the survey spectra of CBP0 and CBP3. Peaks ascribed to C 1s, Bi 4f, Cu 2p, and O 1s are observed in both plots, as expected. Fig. 6C and D show the Bi 4f spectra of both samples. Symmetrical peaks corresponding to Bi 4f_7/2_ and Bi 4f_5/2_ doublets are observed in both samples. In CBP0, this doublet has a distinct shoulder owing to the multiphasic nature of the sample, whereas the peaks are narrower in CBP3. In CBP0, the Bi 4f_7/2_ peak is deconvoluted into two peaks corresponding to the contributions of the CBO (159.26 eV) and α-Bi_2_O_3_ (157.44 eV) phases.^51–53^ Similarly, the Bi 4f_5/2_ peak is deconvoluted peaks corresponding to CBO (164.55 eV) and α-Bi_2_O_3_ (162.81 eV). For CBP3, the deconvoluted Bi 4f_7/2_ (159.28 and 160.4 eV) and Bi 4f_5/2_ (164.58 and 165.72 eV) peaks show presence of Bi^3+^ in the α- and β-Bi_2_O_3_ environments.^54,55^ In the Cu 2p spectra for CBP0 (see Fig. 6E), a doublet of Cu 2p_3/2_ (932.09 eV) and Cu 2p_1/2_ (951.85 eV) is observed. Additionally, two shake-up satellite peaks (941.27 eV and 938.27 eV) are observed, which are characteristic of Cu^2+^ in CuBi_2_O_4_ and CuO.^56^ Similar peaks are observed for the CBP3 (see Fig. 6F), Cu 2p_3/2_ (933.34 eV), and Cu 2p_1/2_ (953.22 eV) samples. Satellite peaks are also present (943.32 and 940.65 eV); however, their intensity relative to the intensity of the doublet is quite low, compared with the intensity for CBP0. This is a characteristic of Cu^1+^ in Cu_2_O, where the satellite peaks are absent or highly diminished.^57^ These observations agree well with the phase analysis based on XRD. In Fig. 6G and H, the O 1s spectra for CBP0 and CBP3 show singlet peaks at 529.52 eV and 531.39 eV, respectively. The components arising from the lattice oxygen, defect oxygen, and chemisorbed oxygen are shown. The oxygen vacancies in CBP3 are evidently higher than in CBP0, which is highly beneficial for photocatalytic applications.

**Table 3:** Peak positions in the XPS spectra

Sr.	XPS spectra	CBP0		CBP3	
		Phases	Binding energy peaks (eV)	Phases	Binding energy peaks (eV)
1	Bi 4f_7/2_	α-Bi_2_O_3_	157.44	β-Bi_2_O_3_	159.28
		CuBi_2_O_4_	159.26	α-Bi_2_O_3_	160.4
	Bi 4f_5/2_	α-Bi_2_O_3_	162.81	β-Bi_2_O_3_	164.58
		CuBi_2_O_4_	164.55	α-Bi_2_O_3_	165.72
2	Cu 2p_3/2_	CuBi_2_O_4_/CuO	932.09	Cu_2_O	933.34
	Cu 2p_1/2_		951.85		953.22
	Satellite 1		941.27		943.32
	Satellite 2		938.27		940.65
3	O 1s		529.52		531.39

**Fig. 6:**
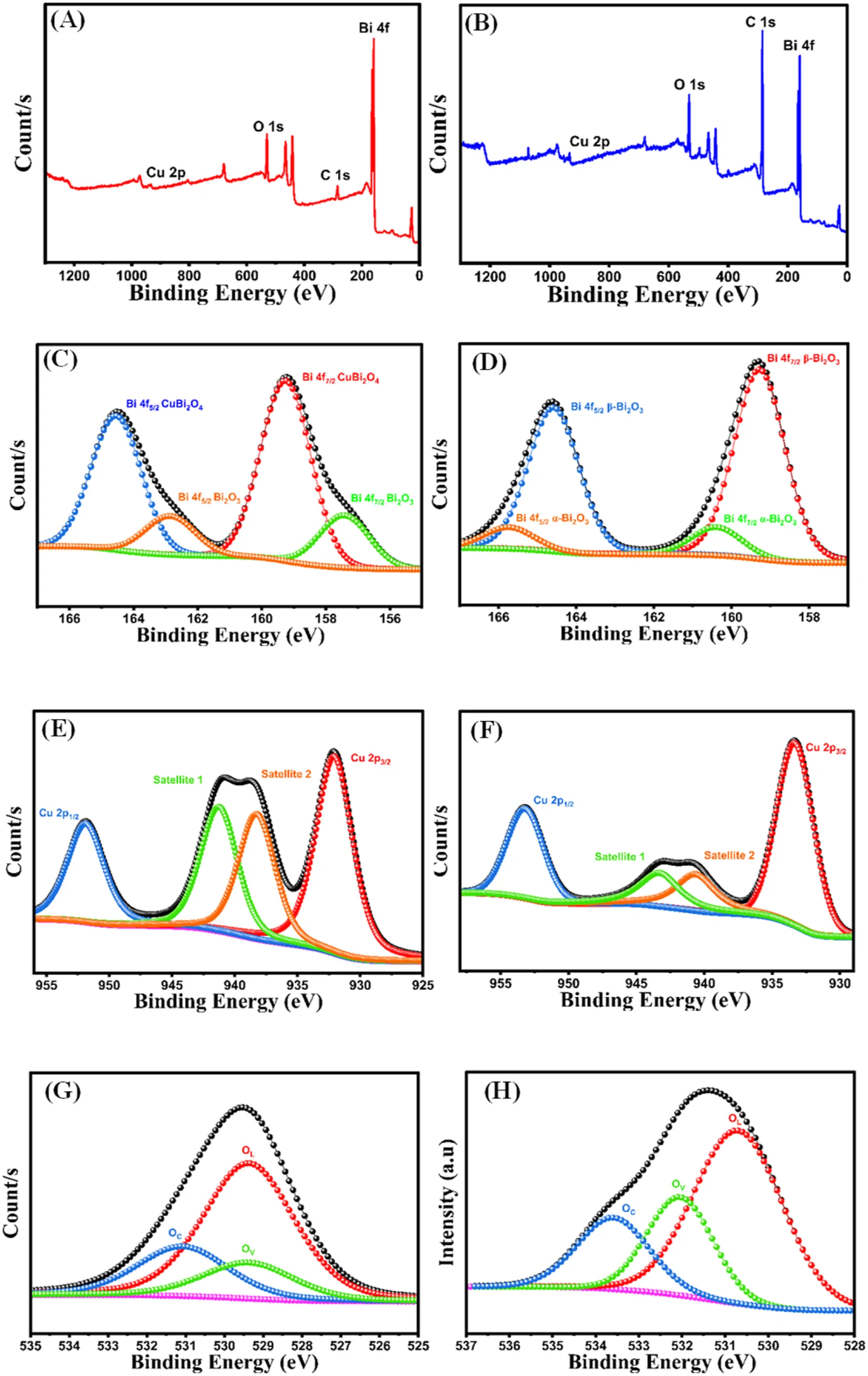
(A) XPS survey of CBP0; (B) XPS survey of CBP3; XPS spectra of CBP0: (C) Bi 4f, (E) Cu 2p, and (G) O 1s; XPS spectra of CBP3: (D) Bi 4f, (F) Cu 2p, and (H) O 1s.

### PEC performance

The chronoamperometry plots are shown in Fig. 7A, and photocurrent values and differences are listed in Table 4. CBP3 sample has the highest transient photocurrent of 9.76 µA, indicating that it has a strong PEC performance. It also exhibits the highest difference between the current densities under light and dark conditions, indicating a high photoactivity. By contrast, CBP0, which was synthesized without PVA, shows a relatively low photocurrent and difference. The photocurrent gradually increases from CBP1 to CBP3 with increasing PVA content, suggesting that moderate PVA addition improves the surface morphology, enhances the active area, and leads to the formation of efficient heterostructures. However, when the PVA content is increased further (CBP4–CBP6), the photocurrent decreases. This is possibly due to impurity phase formation, increased carbon residue, and wider bandgaps (>2.6 eV), which hinder light absorption. CBP6 in particular suffers from the formation of a metallic Bi phase, which acts as an electron trap and reduces performance.^58^ The superior PEC performance of CBP3 to those of the other samples can be attributed to its optimized morphology and favorable band gap of approximately 2.38 eV. *In situ* heterostructure formation, which reduces charge carrier recombination, also increases the performance. These results clearly show that PVA concentration plays a crucial role in tuning the PEC behavior of the mixed-phase sample. CBP3, with its optimal amount of PVA, has emerged as an interesting option for visible-light-driven applications. Subsequently, CBP0 was chosen as the unmodified reference sample, while CBP3 was designated as the representative optimized PVA-mediated sample.

**Fig. 7:**
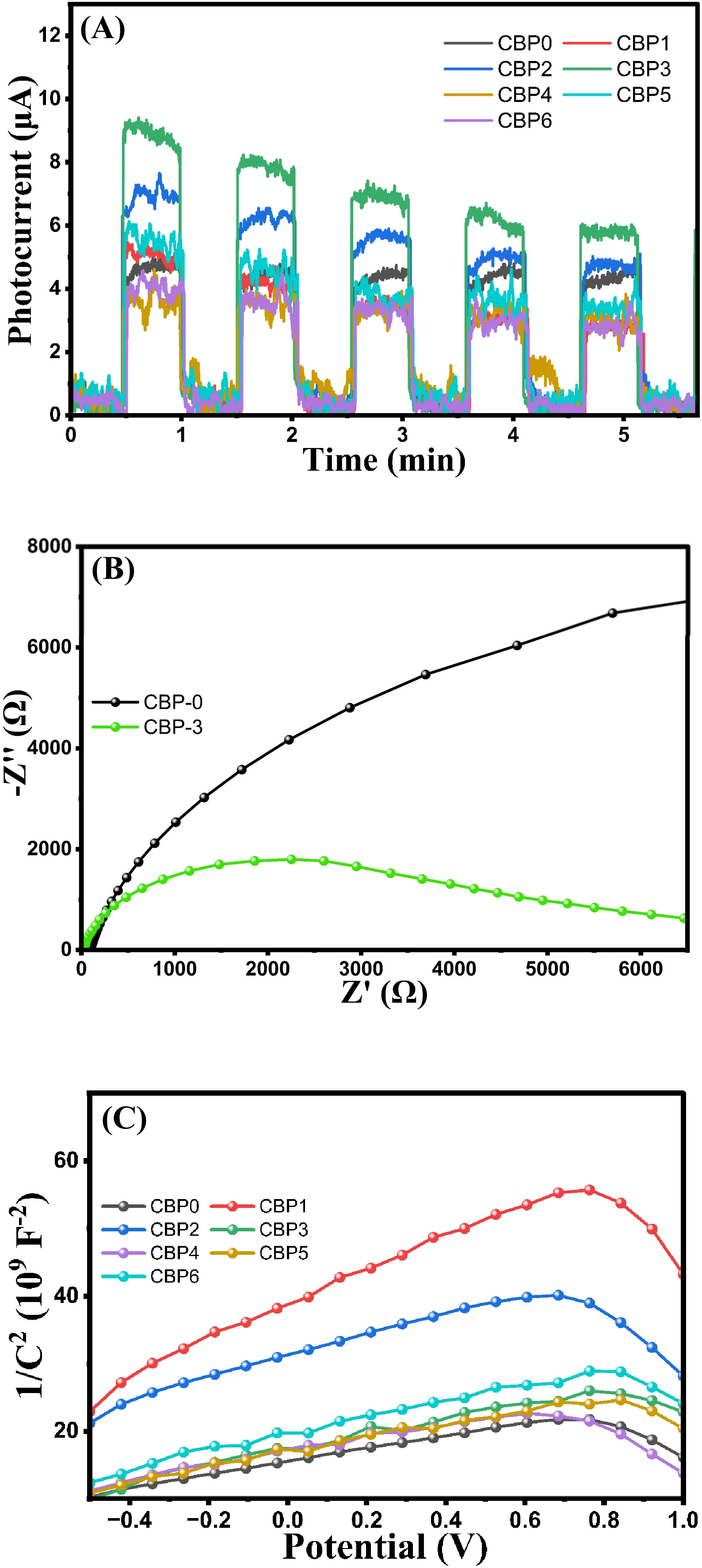
(A) PEC measurements of CBP0–CBP6, (B) EIS plots of CBP0–CBP3, and (C) Mott–Schottky plots of CBP0–CBP6.

**Table 4:** Photocurrent values

Sr. no.	Sample code	Current (light off) (µA)	Photo current (light on) (µA)
1	CBP0	0	5.13
2	CBP1	0	5.62
3	CBP2	0	7.69
4	CBP3	0	9.76
5	CBP4	0	3.69
6	CBP5	0	6.10
7	CBP6	0	4.14

### EIS

EIS was employed to analyze the charge transfer resistance of the synthesized heterostructure electrodes (CBP0 and CBP3). Measurements were performed in dark. As shown in Fig. 7B, the EIS spectra do not display a fully ideal semicircle. This behavior can be linked to the non-ideal and heterogeneous characteristics of the CBO/ITO electrode–electrolyte interface, which include surface heterogeneity, defects, and a range of interfacial relaxation processes. These factors may lead to incomplete semicircular response in the Nyquist plot. Moreover, the impedance response observed in the high-frequency region indicates the resistive and interfacial properties of the heterostructured electrodes. Among the samples examined, CBP3 shows a notably lower impedance response compared to CBP0, indicating more advantageous interfacial charge-transfer characteristics and implying reduced charge-transfer resistance for CBP3. This indicates that when the PVA has introduced during the synthesis, the interfacial conductivity and charge transfer kinetics are improved. PVA enhances the interfacial contact and helps refine the morphology, thereby leading to efficient electron transport. This trend of CBP3 having the lowest charge transfer resistance are consistent with the PEC results, which identifies CBP3 as the most efficient composition.

### Mott–Schottky analysis

Mott–Schottky (M–S) analysis was conducted to investigate the semiconductor type and charge transfer mechanism of the synthesized samples. The M–S plots in Fig. 7C show the relationship between 1/*C*^2^ and the applied potential for each sample (CBP0–CBP6). A characteristic feature of all the plots is an inverted V-shape with n-type behavior at negative potentials that changes to p-type behavior at potentials above 0.8 V. The n-type behavior can be attributed to the α-Bi_2_O_3_ and β-Bi_2_O_3_ phases, whereas the p-type behavior is due to the CBO, CuO, or Cu_2_O phases present in the different samples.^59,60^ The presence of both p- and n-type conductivity indicates the formation of an *in situ* p–n heterojunction in all the samples, which leads to efficient charge carrier separation.^61^ The *in situ* formation of the heterojunction also ensures good contact between the individual components, thereby leading to better charge transfer and higher photoelectrocatalytic efficiency.

### Photoluminescence analysis

To study the charge separation efficiency, steady-state photoluminescence (PL) measurements were performed over a wavelength range of 380–800 nm. Fig. 8A shows that the pristine sample CBP0 exhibits two prominent emission peaks at approximately 450 and 660 nm, attributed to recombination of charge carriers. In contrast, CBP3 exhibits these peaks with a significantly decreased intensity, indicating improved charge separation.^62^ Time-resolved photoluminescence spectra (TRPL) was performed, to understand the lifetimes of photogenerated carriers (Fig. 8B). The average lifetime decreased from 1.56 ns for CBP0 to 0.61 ns for CBP3, indicating faster interfacial charge transfer and more efficient extraction of photogenerated charge carriers in the *in situ* formed Bi_2_O_3_/Cu_2_O heterojunction in CBP3.^63,64^

**Fig. 8:**
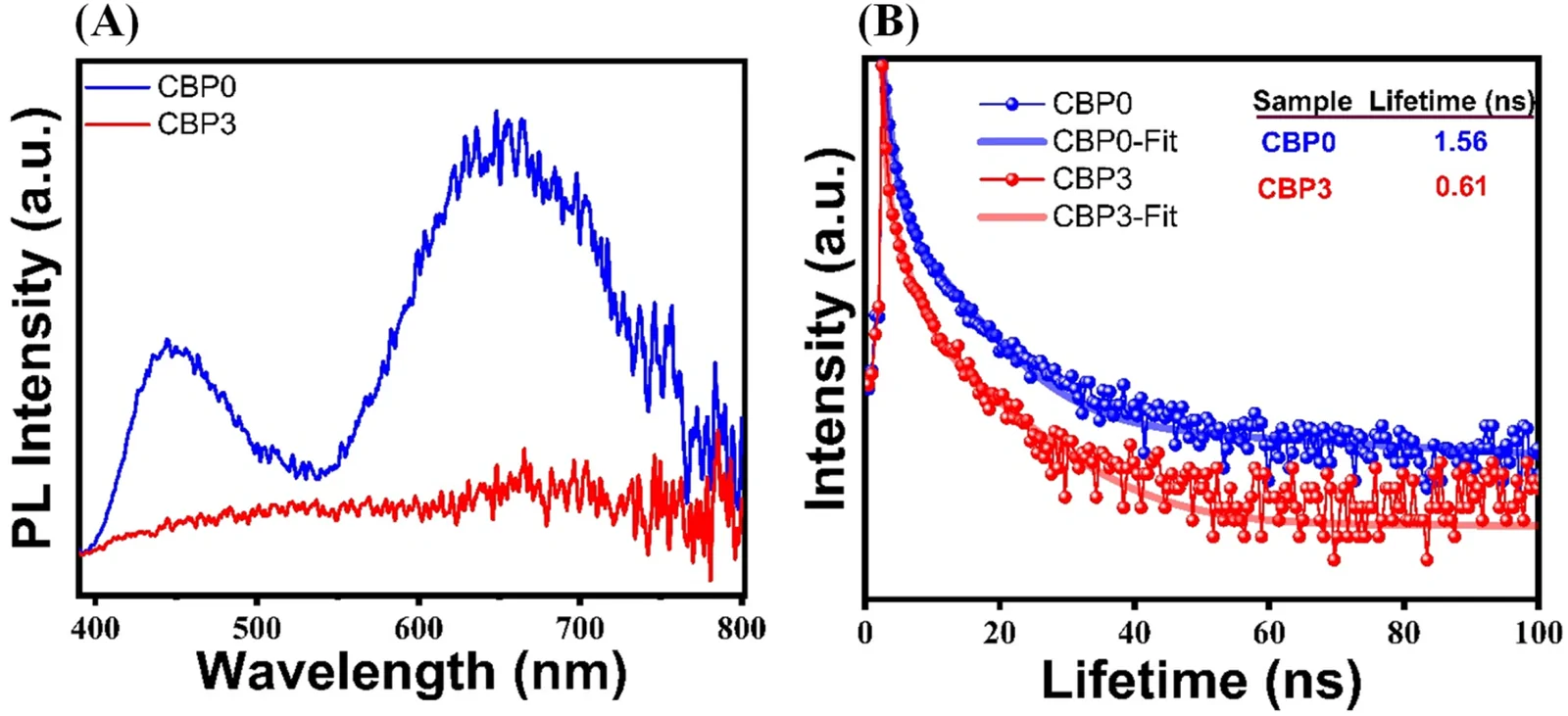
(A) PL measurements of CBP0 and CBP3 (B) TRPL measurements of CBP0 and CBP3.

### Water splitting test

To assess the H_2_ evolution performances of the as-prepared samples, we performed PEC measurements under simulated solar light. As shown in Fig. 9, the samples synthesized with and without PVA exhibit different results. CBP0, which is prepared without PVA, exhibits a relatively low H_2_ evolution of 166 µmol g^−1^, which indicates sluggish charge separation. This is primarily due to its irregular morphology, lower carrier concentration, and higher charge-transfer resistance compared with the other samples. Additionally, presence of the less efficient α-Bi_2_O_3_ and CuO phases probably causes sluggish charge transfer. By comparison, CBP3 shows an outstanding H_2_ evolution of 776 µmol g^−1^, indicating improved water-splitting efficiency. This improvement is linked to the formation of the *in situ* α-Bi_2_O_3_/β-Bi_2_O_3_/Cu_2_O heterostructure due to optimum PVA addition in synthesis. The PVA addition during synthesis improves the particle distribution and enhances the interfacial contact between the heterojunction components. Furthermore, the present H_2_ evolution data are comparable to or exceed those published in the literature for CuBi_2_O_4_, Cu_2_O, and Bi_2_O_3_ based heterostructures (Table TS2). Hence, these results suggest the present catalysts exhibit superior photocatalytic performance.

**Fig. 9:**
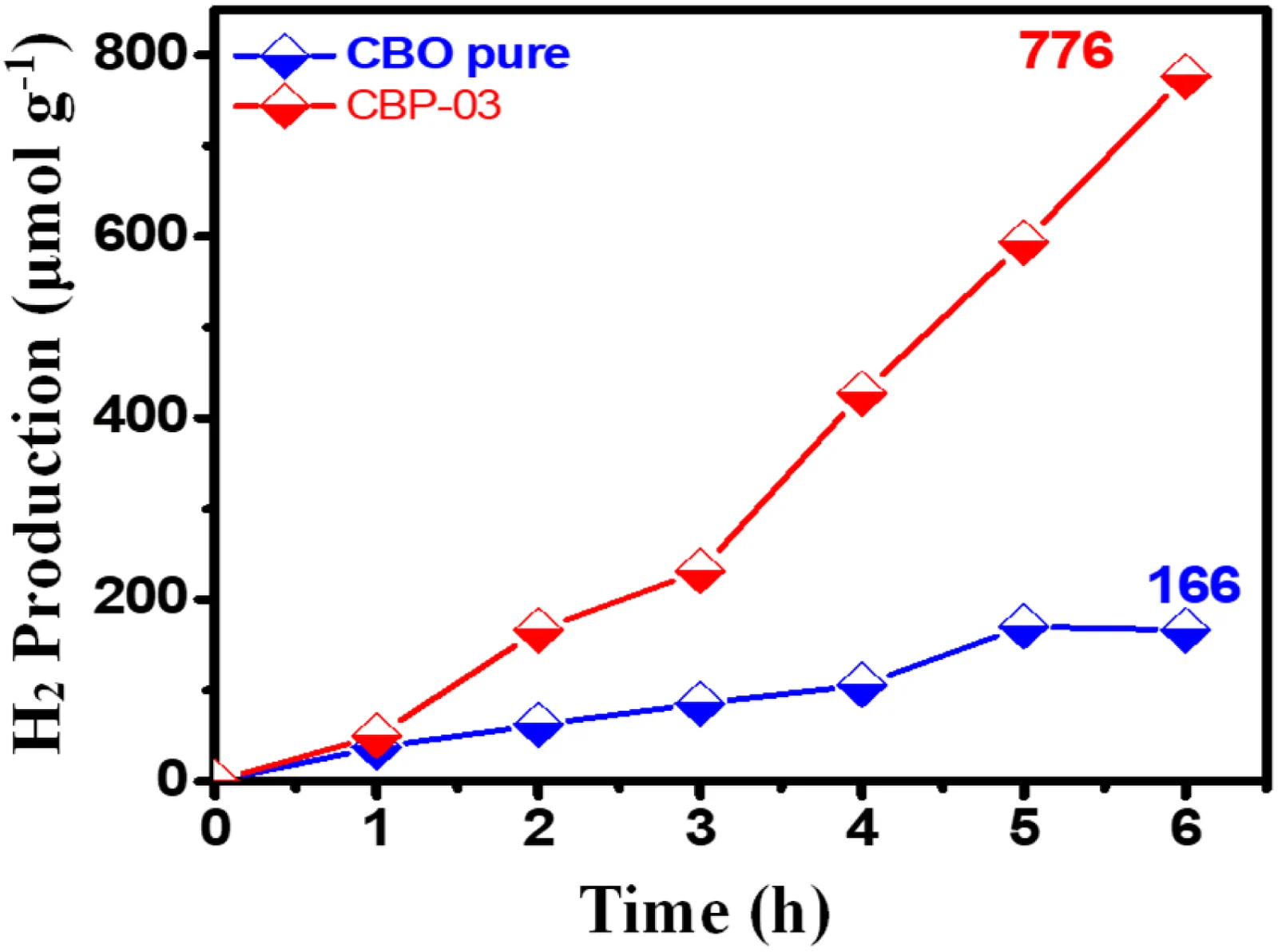
Water-splitting tests of CBP0 and CBP3.

In order to evaluate the reusability of the optimized photocatalyst, we performed the water splitting experiment across four consecutive runs using the CBP3 heterostructure (Fig. S3). It is clear that the hydrogen evolution rate stayed almost stable, maintaining 94.3% of its initial activity, with no substantial decline observed compared to the initial run. As a result, this recycling test indicates that the present CBP3 heterostructure shows stability for practical application. Subsequently, the FESEM and XRD pattern of the CBP3 sample were examined following the stability test. The SEM images (Fig. S4-A) indicate that the morphology exhibits minimal alteration, characterized by slight agglomeration and an increase in pore sizes. The XRD (Fig. S4-B) indicated that the CBP3 sample preserved its crystallinity and exhibited a nearly identical percentage of the Cu_2_O phase following stability testing. However, the percentage of β-Bi_2_O_3_ decreases from 63.8% to 48.5%, while α-Bi_2_O_3_ increases from 21.2% to 37.0%, as indicated by Rietveld refinement (Table TS3). The alteration in the β-Bi_2_O_3_ phase is attributed to its quasi stability at room temperature in contrast to α-Bi_2_O_3_. Interestingly, the total phase percentage of α and β phases of Bi_2_O_3_ remained consistent alongside the Cu_2_O phase in the CBP3 sample. It is evident that the heterostructure exhibits stable characteristics following the recycling tests.

### Possible charge transfer mechanism

CBP0 and CBP3 both samples follow Type-II charge transfer characteristics as provided in the proposed charge transfer mechanism (Fig. S2(A & B)). As, the electrons from Conduction band minima of CuO and CuBi_2_O_4_ can migrate to that of α-Bi_2_O_3_ conduction band minima having less energy (below to CBM of CuO and CuBi_2_O_4_). Although, there is ternary junction, but the conduction band position of α-Bi_2_O_3_ is not as sufficient for H_2_ generation. Apart from this, in CBP3 the electrons from conduction band minima of Cu_2_O goes to conduction band of α/β-Bi_2_O_3_ having sufficient reduction potential and enhanced charge separation, resulting in enhanced water splitting activity. The Cu_2_O phase formation is beneficial because it possesses a more negative conduction band edge, leading to a higher reducing power for H_2_ evolution. The synergistic effects of favorable band alignment, higher carrier density, and lower resistance in the heterostructure make CBP3 a promising candidate for enhancing photocatalytic H_2_ production.

## Conclusions

In summary, this study showed that introducing PVA during the thermal synthesis of CBO fundamentally alters its phase evolution and morphology while significantly enhancing its PEC properties. Without PVA addition, bare CBP0 yielded a primary kusachiite phase with a band gap of 1.62 eV, and it exhibited severe kinetic limitations, which resulted in a low H_2_ evolution rate of only 166 µmol g^−1^. PVA functioned as a structure-directing agent, and at optimal concentration (CBP3) facilitated an *in situ* transformation into a complex α-Bi_2_O_3_/β-Bi_2_O_3_/Cu_2_O heterostructure with a band gap of 2.38 eV. This structural modification drastically improved the charge carrier separation and interfacial conductivity, thereby allowing the optimized CBP3 heterojunction to achieve a maximum photocurrent density of 15.4 µA and an exceptional H_2_ evolution rate of 776 µmol g^−1^. This corresponds an approximately 4.7-fold enhancement in the PEC water splitting activity compared to the pristine CBO structure, thereby positioning the CBP3 heterojunction as a highly efficient photocatalyst.

## Author contributions

Indu Kumari conceived the study, carried out the synthesis, experimental work and wrote the first draft of manuscript. Hazina Charles, Plassidius J. Chengula and Vijay Kumar performed characterizations and photocatalytic measurement. Siddhant Deshpande contributed in writing the manuscript. Caroline S. Lee and Rajendra C. Pawar supervised the research work, contributed to data interpretation and critically revised the manuscript. All authors reviewed and approved the final manuscript.

## Conflicts of interest

The authors declare that they have no known competing financial interests or personal relationships that could have appeared to influence the work reported in this paper.

## Supplementary Material

RA-016-D6RA07504E-s001

## Data Availability

The data supporting the findings of this study are available within the article and from the corresponding author upon reasonable request. Supplementary information (SI) is available. See DOI: https://doi.org/10.1039/d6ra07504e.
