# Supplementary material for: In situ transformation of CuBi2O4 to heterogeneous Bi2O3/Cu2O mixed phase using thermal method for exceptional H2 production
Source: RSC Adv. 2026 Oct 23;16(48):65528–39. doi: 10.1039/d6ra07504e (PMC13692006; doi:10.1039/d6ra07504e)
Supplement: Supplementary file 1 [file RA-016-D6RA07504E-s001.pdf]

Supplementary Information (SI) for RSC Advances.
This journal is © The Royal Society of Chemistry

Supporting Information

Supporting Figure Captions

Figure S1. Rietveld Refinement XRD Graphs of samples; (A) CBP0, (B) CBP1, (C)CBP2, (D)CBP3, (E) CBP4, (F) CBP5 and (G) CBP6.

Figure S2. Proposed Charge Transfer mechanism: (A) CBP0 and (B) CBP3

Figure S3. Stability test for water splitting of CBP3 sample

Figure S4. SEM images (A: I, II & III) and XRD pattern (B) of CBP3 sample after four consecutive H_2 evolution stability cycles

Supporting Table Captions

Table TS1. Phase Composition of different phases for various prepared samples

Table TS2. Comparison with recently reported Literature

Table TS3 Comparison of phase composition of CBP3 sample before and after water splitting and stability tests.

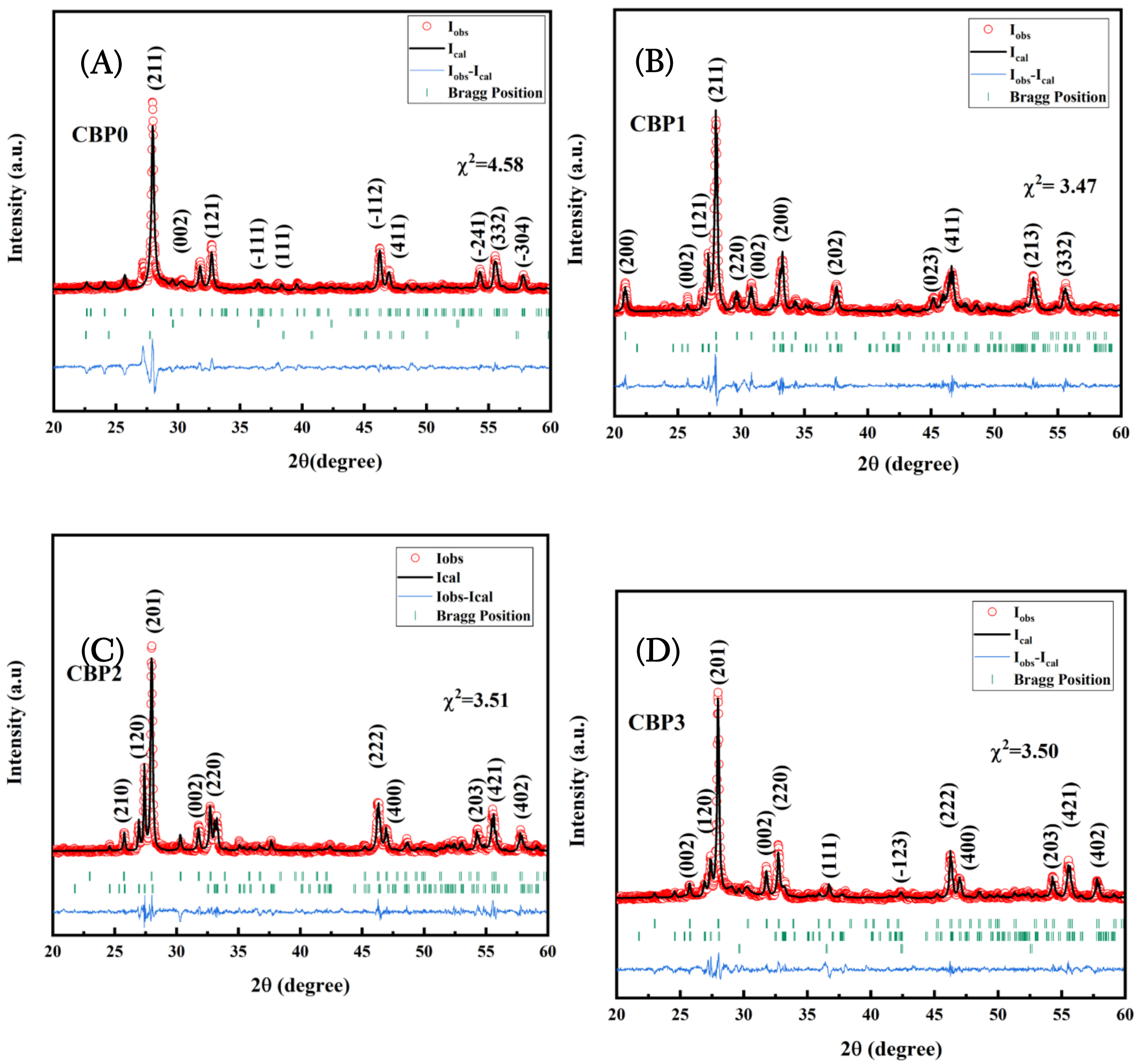

(A)
CBP0
Iobs
Ical
Iobs-Ical
Bragg Position
χ2=4.58
(211)
(002)
(121)
(-111)
(111)
(-112)
(411)
(-241)
(332)
(-304)
Intensity (a.u.)
2θ(degree)
20 25 30 35 40 45 50 55 60
(B)
CBP1
Iobs
Ical
Iobs-Ical
Bragg Position
χ2= 3.47
(200)
(002)
(121)
(211)
(220)
(002)
(200)
(202)
(023)
(411)
(213)
(332)
Intensity (a.u.)
2θ (degree)
20 25 30 35 40 45 50 55 60
(C)
CBP2
Iobs
Ical
Iobs-Ical
Bragg Position
χ2=3.51
(210)
(120)
(201)
(002)
(220)
(222)
(400)
(203)
(421)
(402)
Intensity (a.u)
2θ (degree)
20 25 30 35 40 45 50 55 60
(D)
CBP3
Iobs
Ical
Iobs-Ical
Bragg Position
χ2=3.50
(002)
(120)
(201)
(002)
(220)
(111)
(-123)
(222)
(400)
(203)
(421)
(402)
Intensity (a.u.)
2θ (degree)
20 25 30 35 40 45 50 55 60

Figure S1(A to D)

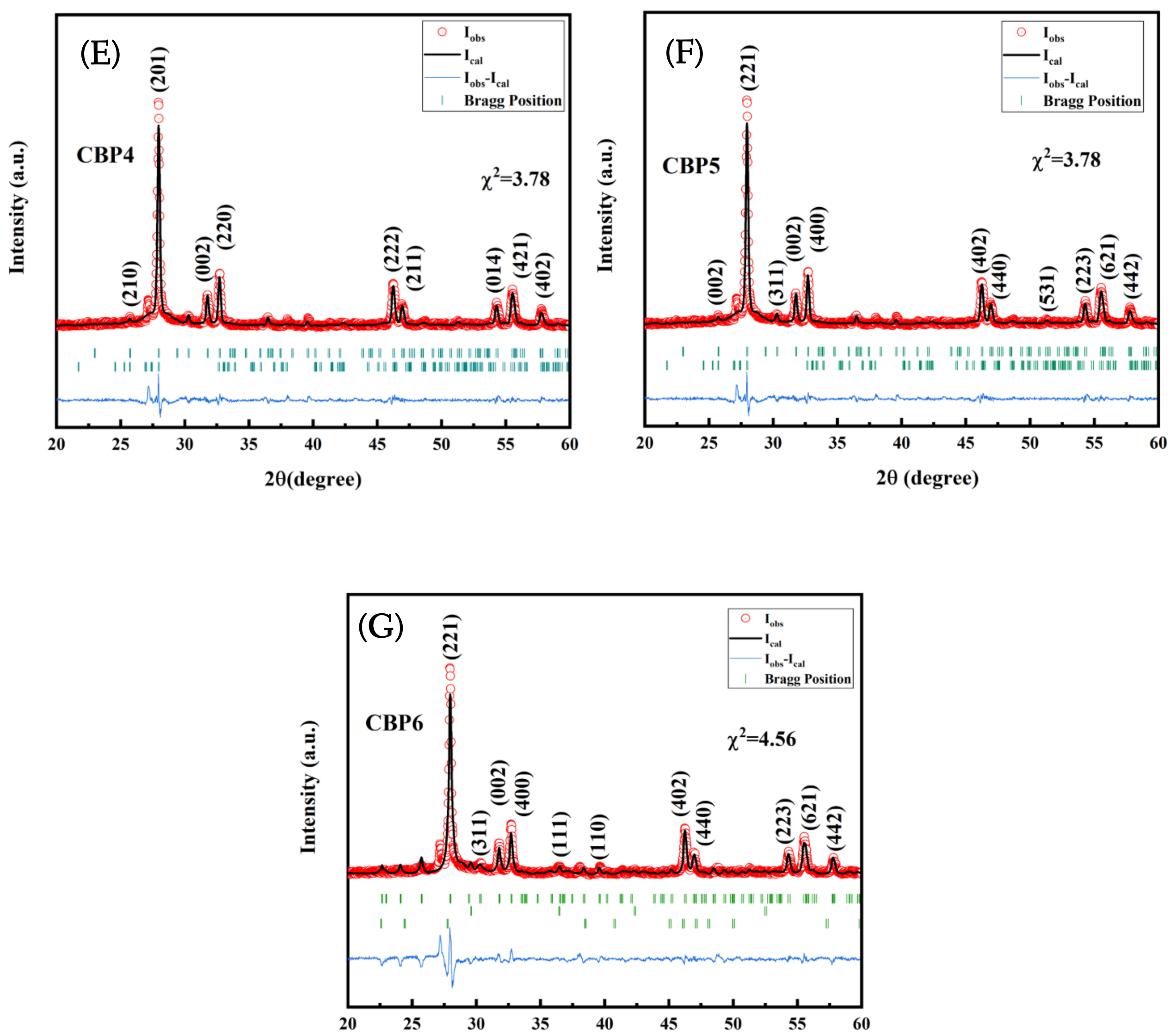

Figure S1(E to G)

(A)

(B)

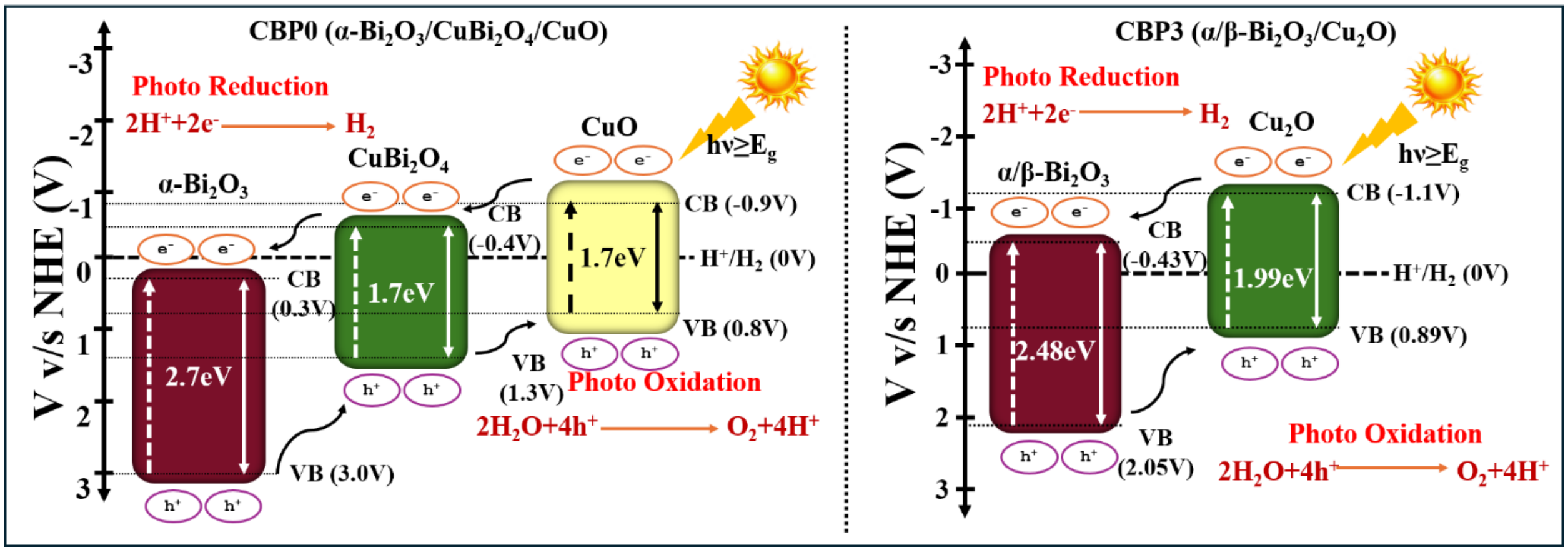
CBP0 (α-Bi2O3/CuBi2O4/CuO)
Photo Reduction
2H++2e- → H2
α-Bi2O3
CuBi2O4
CuO
hν≥Eg
CB (-0.9V)
CB (-0.4V)
CB (0.3V)
1.7eV
1.7eV
2.7eV
H+/H2 (0V)
VB (0.8V)
VB (1.3V)
VB (3.0V)
Photo Oxidation
2H2O+4h+ → O2+4H+
V v/s NHE (V)
CBP3 (α/β-Bi2O3/Cu2O)
Photo Reduction
2H++2e- → H2
Cu2O
hν≥Eg
α/β-Bi2O3
CB (-1.1V)
CB (-0.43V)
1.99eV
2.48eV
H+/H2 (0V)
VB (0.89V)
VB (2.05V)
Photo Oxidation
2H2O+4h+ → O2+4H+
V v/s NHE (V)

Figure S2 (A & B)

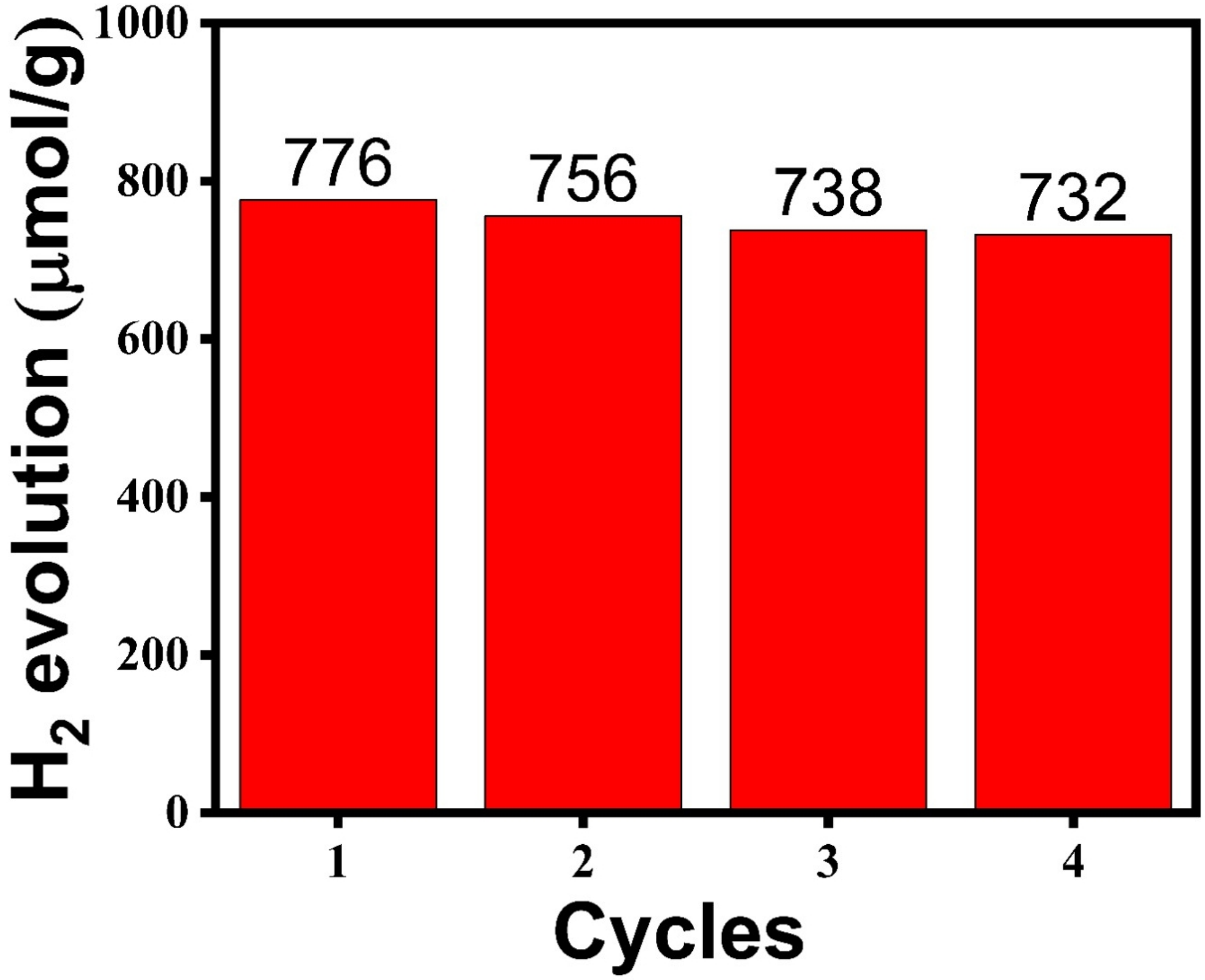

1000
800
600
400
200
0
776
756
738
732
1
2
3
4
Cycles
H2 evolution (μmol/g)

Figure S3

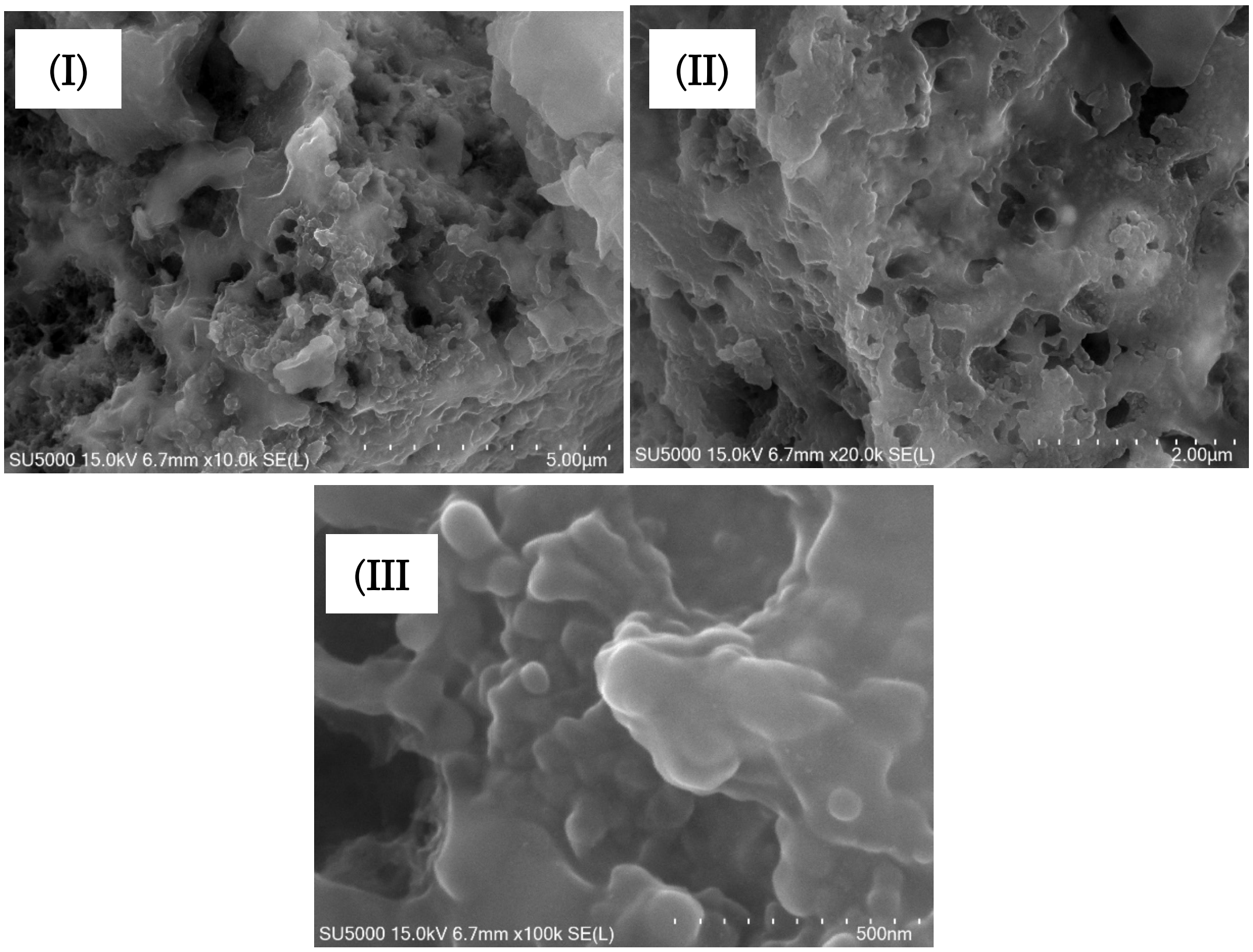
(I)
SU5000 15.0kV 6.7mm x10.0k SE(L)
5.00µm
(II)
SU5000 15.0kV 6.7mm x20.0k SE(L)
2.00µm
(III
SU5000 15.0kV 6.7mm x100k SE(L)
500nm

Figure S4 (A)

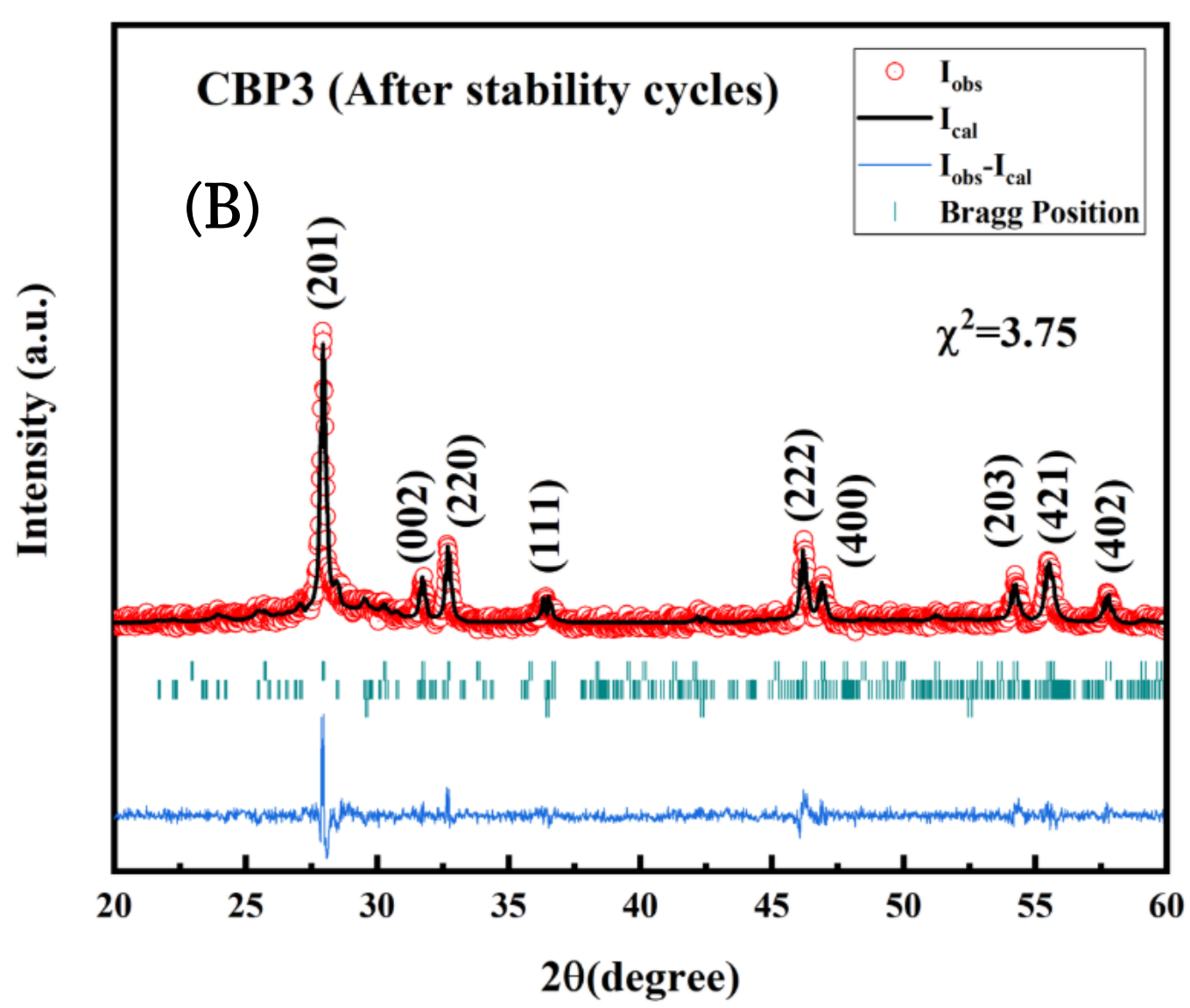
CBP3 (After stability cycles)
(B)
I_{obs}
I_{cal}
I_{obs}-I_{cal}
Bragg Position
χ^2=3.75
(201)
(002)
(220)
(111)
(222)
(400)
(203)
(421)
(402)
Intensity (a.u.)
20
25
30
35
40
45
50
55
60
2θ(degree)

Figure S4(B)

Sr. No.	Sample	χ²	Phase Composition
1	CBP0	4.58	$CuBi_2O_4$=48.4%<br>α-Bi_2O_3=30.7%<br>CuO=20.9%
2	CBP1	3.47	$CuBi_2O_4$=77.9%<br>β-Bi_2O_3=22.1%
3	CBP2	3.51	β-Bi_2O_3=60.2%<br>α-Bi_2O_3=39.8%
4	CBP3	3.50	β-Bi_2O_3=63.8%<br>α-Bi_2O_3=21.2%<br>Cu_2O=14.9%
5	CBP4	3.78	β-Bi_2O_3=80.3%<br>α-Bi_2O_3=19.7%
6	CBP5	3.78	α-Bi_2O_3=22.9%<br>β-Bi_2O_3=77.1%
7	CBP6	4.56	β-Bi_2O_3=85.6%<br>Cu_2O=7.9%<br>Bi=6.5%
8	CBP3 (After four stability cycles)	3.75	α-Bi_2O_3=37.1%<br>β-Bi_2O_3=48.5%<br>Cu_2O=14.5%

Table TS1

Sr. No.	Photocatalyst	Heterojunction Type	Light Source	H_2 Yield	Stability	Ref.

1	$CuBi_2O_4/CuO$	S-scheme	simulated AM 1.5G illumination	7.5 μmol in 30min	-	S1
2	$CuBi_2O4/Bi_2O_3$	Z-scheme	Natural Sunlight	526 μmolg^{-1}h^{-1}	Relatively stable for 24h	S2
3	Carbon Quantum dots mediated $CuBi_2O_4/CuO$	Z-scheme	AM 1.5G Solar Simulator	18.6μmolcm^{-2}h^{-1}	-	S3
4	$CuBi_2O_4$	-	100MWcm^{-2}	4.3mmol in 6 hours	Relatively Stable for 12 hours	S4
5	$CuBi_2O_4/TiO_2$ NTAs		300W Xe Lamp (λ>420nm)	87.42 μmolcm^{-2}h^{-1}	maintains 95.44 % after successive 480 min irradiation	S5
6	NiO/Ag-doped $CuBi_2O_4$	-	150W solar simulator with 1.5G Filter	35 6μmolcm^{-2} In about 2 hours	Stability for 2 hours despite 20% decrease in photocurrent	S6
7	Ag/Bi_2O_3/rGO	Type-II	Visible Light irradiation	432.7μmolg^{-1}h^{-1}	-	S7
8	α/β Bi_2O_3/Cu_2O	Type-II	300W Xe Arc Lamp	776 μmolg^{-1} in 6h	Retained 94.3% of activity after 4th cycle	**This work**

Table TS2

Sr. No.	Sample	$\square^2$	Phase Composition
1	CBP3 (Before water splitting)	3.50	α-Bi_2O_3=21.2%<br>β-Bi_2O_3=63.8%<br>Cu_2O=14.9%
2	CBP3 (After four stability cycles)	3.75	α-Bi_2O_3=37.1%<br>β-Bi_2O_3=48.5%<br>Cu_2O=14.5%

Table TS3

Supporting References:

S1 H. Xiu, F. Fan, P. An, M. Zhang, Y. Wang, Y. Cui, Y. Wang, G. Jiang and C. Xu, Bioinspired hierarchical nanostructured $CuBi_2O_4$/CuO heterojunction photocathode for enhanced performance in photoelectrochemical water splitting, *Green Energy Environ.*, 2026, **11**, 1146–1157.

S2 S. Mondal, L. Patra, P. Ilanchezhiyan, B. Neppolian, R. Pandey and V. Ganesh, In Situ Growth of $CuBi_2O_4$/Bi_2O_3 Z-Scheme Heterostructures for Bifunctional Photocatalytic Applications, *Langmuir*, 2024, **40**, 12954–12966.

S3 Q. Wang, J. Jia, J. Liu and J. Yang, Carbon-Quantum-Dot-Mediated Z-Scheme Charge Transfer in Vertically Aligned $CuBi_2O_4$/CuO Photocathodes for Enhanced Hydrogen Evolution, *ChemCatChem*, 2025, **17**, 1–9.

S4 B. Meena, M. Kumar, R. K. Hocking, S. Juodkazis, V. Biju, P. Subramanyam and C. Subrahmanyam, Exploring $CuBi_2O_4$ as a Promising Photocathode Material for PEC Water Splitting, *Energy and Fuels*, 2023, **37**, 14280–14289.

S5 F. Zhang, Y. Sun, M. Li, Q. Wang, W. Song, J. Ma and J. Hou, Solvothermal preparation of hydrangea-like $CuBi_2O_4$ twining TiO_2 NTAs with enhanced photoelectrocatalytic dye degradation and hydrogen generation, *Colloids Surfaces A Physicochem. Eng. Asp.*, 2023, **667**, 131389.

S6 M. Gopannagari, K. A. J. Reddy, S. Inae, H. S. Bae, J. Lee, T. G. Woo, A. P. Rangappa, D. P. Kumar, D. A. Reddy and T. K. Kim, High-Performance Silver-Doped Porous $CuBi_2O_4$ Photocathode Integrated with NiO Hole-Selective Layer for Improved Photoelectrochemical Water Splitting, *Adv. Sustain. Syst.*, 2023, **7**, 1–11.

S7 P. C. Nethravathi, M. V. Manjula, S. Devaraja, M. Sakar and D. Suresh, Eco-friendly preparation of Bi_2O_3, Ag-Bi_2O_3 and Ag-Bi_2O_3-rGO nanomaterials and their photocatalytic H_2 evolution, dye degradation, nitrite sensing and biological applications, *J. Photochem. Photobiol. A Chem.*, 2023, **435**, 114295.
